# From Dyakonov-Cherenkov radiation to Dyakonov surface optics

**DOI:** 10.1038/s41377-021-00692-6

**Published:** 2022-01-04

**Authors:** Mikhail Dyakonov

**Affiliations:** University of Montpellier, Montpellier, France

**Keywords:** Atom optics, Microwave photonics

**Fig. 1:**
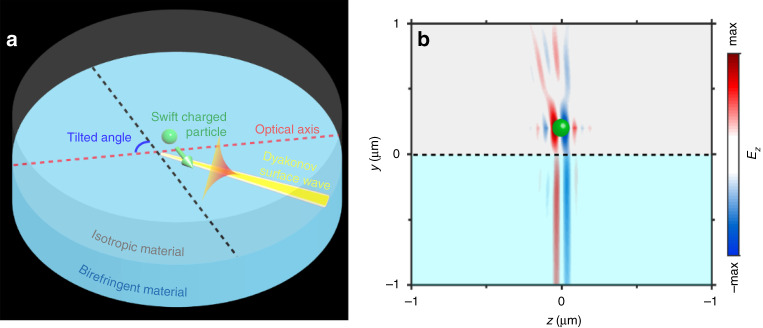
Surface Dyakonov-Cherenkov radiation. **a** Schematics of surface Dyakonov-Cherenkov radiation. A swift charged particle moving in a structure made of semi-infinite isotropic and birefringent material excites Dyakonov surface waves, leading to surface Dyakonov-Cherenkov radiation. **b** Radiation pattern of surface Dyakonov-Cherenkov radiation. (Reproduced with permission from ref. 7 Copyright 2021, Nature Publishing Group.)

Cherenkov radiation by swift charged particles in a dielectric medium has been used for a long time as an effective means for particle detection and measurements of their energy^1–5^. Now a team led by Prof. Yu Luo from Singapore considers a situation when the particle emits surface electromagnetic waves at the interface between an isotropic and a uniaxial-birefringent medium (Dyakonov surface waves^6^). The resulting Dyakonov-Cherenkov radiation is shown to be highly sensitive to both the value and the direction of the particle velocity^7^. In particular, it is shown that close to the Cherenkov threshold, the radiation intensity can be several orders of magnitude greater than that in traditional Cherenkov detectors. These new features allow to determine simultaneously the magnitude and direction of particle velocities on a compact platform (Fig. 1).

On the one hand, this work shows how promising Dyakonov surface waves can be for designing high-sensitivity Cherenkov detectors on-chip; on the other hand, this work proves that the delocalized nature of free charged particles can provide an efficient approach to excite Dyakonov surface waves, opening the door to transplant useful applications of surface plasmon optics to Dyakonov surface optics. In particular, Dyakonov surface waves can be excited in an all-dielectric platform, and hence, they generally have longer propagation lengths than the well known surface plasmons, even at low frequencies. This advantage makes Dyakonov surface waves an alternative platform for future on-chip communications at microwave and millimeter-wave frequencies^8^.
