# Correction: From Dyakonov-Cherenkov radiation to Dyakonov surface optics

**DOI:** 10.1038/s41377-022-00770-3

**Published:** 2022-04-07

**Authors:** Mikhail Dyakonov

**Affiliations:** University of Montpellier, Montpellier, France

**Keywords:** Atom optics, Microwave photonics

Correction to: *Light: Science & Applications*

10.1038/s41377-021-00692-6 published online 04 January 2022

In the version of this article^1^ originally published, the author Mikhail Dyakonov’s affiliation was mistaken. Now the author’s affiliation is corrected to below:

University of Montpellier, Montpellier, France

The original article has been updated.
